# ADCC Develops Over Time during Persistent Infection with Live-Attenuated SIV and Is Associated with Complete Protection against SIV_mac_251 Challenge

**DOI:** 10.1371/journal.ppat.1002890

**Published:** 2012-08-23

**Authors:** Michael D. Alpert, Jackson D. Harvey, W. Anderson Lauer, R. Keith Reeves, Michael Piatak, Angela Carville, Keith G. Mansfield, Jeffrey D. Lifson, Wenjun Li, Ronald C. Desrosiers, R. Paul Johnson, David T. Evans

**Affiliations:** 1 Department of Microbiology and Immunobiology, Harvard Medical School, New England Primate Research Center, Southborough, Massachusetts, United States of America; 2 Immunology Division, Harvard Medical School, New England Primate Research Center, Southborough, Massachusetts, United States of America; 3 SAIC Frederick, National Cancer Institute at Frederick, Frederick, Maryland, United States of America; 4 Department of Pathology, Harvard Medical School, New England Primate Research Center, Southborough, Massachusetts, United States of America; 5 University of Massachusetts Medical School, Worcester, Massachusetts, United States of America; 6 Ragon Institute of Massachusetts General Hospital, MIT, and Harvard, and Infectious Disease Unit, Massachusetts General Hospital, Boston, Massachusetts, United States of America; University of Zurich, Switzerland

## Abstract

Live-attenuated strains of simian immunodeficiency virus (SIV) routinely confer apparent sterilizing immunity against pathogenic SIV challenge in rhesus macaques. Understanding the mechanisms of protection by live-attenuated SIV may provide important insights into the immune responses needed for protection against HIV-1. Here we investigated the development of antibodies that are functional against neutralization-resistant SIV challenge strains, and tested the hypothesis that these antibodies are associated with protection. In the absence of detectable neutralizing antibodies, Env-specific antibody-dependent cell-mediated cytotoxicity (ADCC) emerged by three weeks after inoculation with SIVΔ*nef*, increased progressively over time, and was proportional to SIVΔ*nef* replication. Persistent infection with SIVΔ*nef* elicited significantly higher ADCC titers than immunization with a non-persistent SIV strain that is limited to a single cycle of infection. ADCC titers were higher against viruses matched to the vaccine strain in Env, but were measurable against viruses expressing heterologous Env proteins. In two separate experiments, which took advantage of either the strain-specificity or the time-dependent maturation of immunity to overcome complete protection against SIV_mac_251 challenge, measures of ADCC activity were higher among the SIVΔ*nef*-inoculated macaques that remained uninfected than among those that became infected. These observations show that features of the antibody response elicited by SIVΔ*nef* are consistent with hallmarks of protection by live-attenuated SIV, and reveal an association between Env-specific antibodies that direct ADCC and apparent sterilizing protection by SIVΔ*nef*.

## Author Summary

Live-attenuated vaccines can prevent simian immunodeficiency virus (SIV) infection upon experimental challenge of rhesus macaques. Although safety considerations preclude vaccinating humans with live-attenuated HIV-1, it may be possible to replicate the types of immunity induced by live-attenuated SIV through an alternative approach. Thus, identifying the immune responses underlying protection by live-attenuated SIV and understanding their induction would provide guidance for HIV-1 vaccine design. An important role for the maturation of virus-specific antibody responses could explain the time-dependent development of protection by live-attenuated SIV. However, antibodies that block the entry of the challenge virus into cells are usually undetectable. Antibodies can also direct the killing of virus-infected cells by antibody-dependent cell-mediated cytotoxicity (ADCC). Here we show that live-attenuated SIV induces progressive increases in ADCC over time, and that the development of these antibodies is dependent upon the persistent replication of the vaccine strain. In two different experiments, the animals immunized with live-attenuated SIV that remained uninfected after pathogenic SIV challenge had higher measures of ADCC than those that became infected. Our results suggest that antibodies contribute to protection by live-attenuated SIV, and that persistent stimulation of antibody responses may be essential for HIV-1 vaccines to induce high ADCC activity.

## Introduction

The development of a vaccine against HIV-1 continues to be hampered by our limited understanding of the types of immune responses needed for protection. Although safety considerations preclude the use of live-attenuated HIV-1 in people [1]–[5], live-attenuated strains of simian immunodeficiency virus (SIV) afford the most reliable protection achieved to date in non-human primate models, often providing apparent sterilizing immunity against closely related challenge viruses [6]–[9]. Thus, identifying the immune responses that mediate protection by live-attenuated SIV and understanding how to elicit them by vaccination may provide important insights for the development of a safe and effective HIV-1 vaccine [10].

Antibody, T cell, and innate immunity have evolved to operate synergistically as an integrated system [11]–[13], and a combination of these immune responses may be necessary for complete protection by live-attenuated SIV. However, the efficacy of at least one of these immune responses increases over time, since animals challenged with pathogenic SIV_mac_251 months after inoculation with live-attenuated SIV are protected from infection, whereas animals challenged at early time points become infected [7], [8]. Although live-attenuated SIV elicits virus-specific T cells [14]–[16], and the quality of these T cell responses may change over time, the frequency of virus-specific CD8^+^ T cells declines after the acute peak of live-attenuated SIV replication [17]. In contrast, antibodies capable of neutralizing virus infectivity develop over time through affinity maturation [18]–[21]. An essential role for the affinity maturation of antibody responses could account for the time-dependent development of protection by live-attenuated SIV [22]. However, SIV_mac_251 is inherently resistant to neutralization [23], and antibodies capable of neutralizing this challenge virus are typically undetectable among completely protected animals [8], [9]. We therefore reasoned that functions of antibodies other than neutralization may contribute to protection by live-attenuated SIV.

In addition to virus neutralization, the antiviral functions of antibodies include complement fixation and numerous consequences of Fc receptor crosslinking, such as ADCC [13], [24]–[29]. Since ADCC represents a potential effector mechanism and a proxy for other activities of the same antibodies, we developed a novel assay for quantifying the ability of antibodies to direct ADCC. This assay measures ADCC against virus-infected target cells expressing native conformations of the viral envelope glycoprotein (Env), and is therefore more physiologically relevant than methods based on coating target cells with recombinant gp120, gp140, or peptides [30]–[37]. We used this assay to investigate the induction of antibodies with ADCC activity, and to test the hypothesis that higher ADCC activity against cells infected by the challenge virus is associated with protection. Our results indicate that persistent infection with SIVΔ*nef* elicits Env-specific ADCC titers that develop over time, are cross-reactive with Env proteins expressed by heterologous SIV strains, are proportional to vaccine strain replication, and are higher among animals protected against SIV_mac_251 infection.

## Results

### Time-dependent maturation of antibody responses

**Figure 1:**
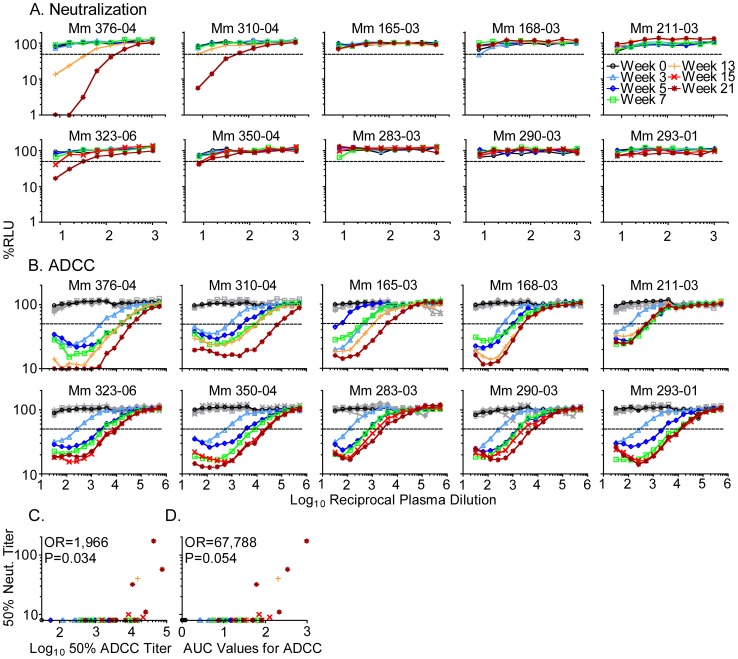
Development of neutralizing antibody and ADCC titers in macaques inoculated with SIV_mac_239Δ*nef*. Plasma collected from 10 animals at 0, 3, 5, 7, 13 or 15, and 21 weeks after inoculation with SIV_mac_239Δ*nef* was evaluated for its capacity to neutralize SIV_mac_239 (A) and to direct ADCC against SIV_mac_239-infected cells (B). The loss of relative light units (RLU) indicates the loss of virus-infected cells during an 8-hour incubation in the presence of plasma and an NK cell line. Target cells infected by SHIV_SF162P3_ served as a negative control for all ADCC assays (gray). Dashed lines indicate 50% activity. Neutralizing antibody titers were compared with 50% ADCC titers (C), and with AUC values for ADCC (D). An odds ratio (OR) for the probability of detecting neutralization per log_10_ increase in 50% ADCC titer, or per 1 AUC unit increase in ADCC activity, was estimated by logistic regression.

Plasma samples collected at longitudinal time points after inoculation with SIV_mac_239Δ*nef* were tested for their ability to neutralize SIV_mac_239 and to direct ADCC against SIV_mac_239-infected cells. Only four of ten macaques developed neutralizing antibody titers, and these were not detectable until thirteen weeks after inoculation with SIV_mac_239Δ*nef* (Figure 1A). In contrast, ADCC titers were detectable in all animals just three weeks after inoculation with SIV_mac_239Δ*nef* (Figure 1B). These ADCC titers were Env-specific, since none of the plasma samples had detectable ADCC activity against target cells infected with SHIV_SF162P3_, which expresses the Env protein of HIV-1_SF162_. To quantify ADCC titers, we calculated the plasma dilution that reduces the luciferase signal from virus-infected cells by 50%, and to measure differences in the extent of target cell elimination over all dilutions tested, we calculated values for the area under the curve (AUC). By both measures, progressive increases in ADCC were observed over 21 weeks. Thus, antibody titers capable of directing ADCC against SIV_mac_239-infected cells increased over time, but unlike neutralizing antibodies, emerged early and were detectable in all animals.

Neutralizing antibodies were only detectable in the plasma samples with high ADCC titers. A 50% ADCC titer of approximately 10^4^ emerged as a threshold, below which neutralization of SIV_mac_239 was not detectable (Figure 1C). Among all of the plasma samples collected after inoculation with SIV_mac_239Δ*nef*, the odds of detecting neutralization were 1,966-fold higher per log_10_ increase in 50% ADCC titer (95% CI = 1.8 to 2,192,451, P = 0.034), as estimated by logistic regression (Figure 1C). Likewise, there was a trend towards a higher probability of detecting neutralization by plasma with higher AUC values for ADCC (odds ratio: 67,788-fold higher per 1 AUC unit, 95% CI = 0.835–5.5×10^9^, P = 0.054) (Figure 1D). ADCC titers therefore predicted and were correlated with neutralization.

### Persistent replication required to elicit high ADCC titers

**Figure 2:**
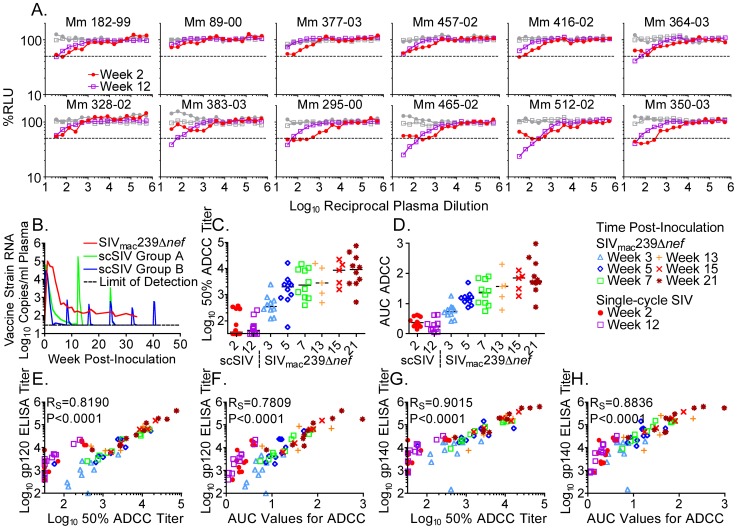
ADCC titers elicited by SIV_mac_239Δ*nef* versus single-cycle SIV. Plasma samples collected on weeks 2 and 12 after a series of inoculations with single-cycle SIV were titered for ADCC against SIV_mac_239-infected cells (A). Target cells infected with SHIV_SF162P3_ served as a negative control (gray). Dashed lines indicate 50% activity. Geometric mean vaccine strain viral loads reflecting virus particles produced *in vivo* after inoculation with SIV_mac_239Δ*nef* or single-cycle SIV are shown (B). Animals in Group A were inoculated 3 times with single-cycle SIV that was *trans*-complemented with the vesicular stomatitis virus glycoprotein (VSV G), whereas the animals in Group B were inoculated 6 times with single-cycle SIV that was not *trans*-complemented [38]. The 50% ADCC titers (C) and the AUC values for ADCC (D) elicited by SIV_mac_239Δ*nef* were significantly higher than those elicited by single-cycle SIV (2-tailed Mann-Whitney U tests, P = 0.0062 to P<0.0001). Binding titers measured by ELISA against SIV_mac_239 gp120 were correlated with 50% ADCC titers (E), and with AUC values for ADCC (F). Binding titers against SIV_mac_239 gp140 were also correlated with 50% ADCC titers (G), and with AUC values for ADCC (H).

The contribution of ongoing vaccine strain replication to the development of antibody responses was evaluated by comparing ADCC in animals immunized with SIV_mac_239Δ*nef* to ADCC in animals immunized with an SIV strain that is limited to a single cycle of infection. Plasma samples collected two or twelve weeks after a series of inoculations with single-cycle SIV [38] were tested for ADCC against SIV_mac_239-infected cells (Figure 2A). Since the geometric mean peak viral RNA loads in plasma for SIV_mac_239Δ*nef* and single-cycle SIV were within two-fold of each other, 1.3×10^5^ and 7.4×10^4^ copies per ml respectively (Figure 2B), differences in antibody responses relate to differences in the persistence of SIV_mac_239Δ*nef* versus single-cycle SIV. Five weeks after inoculation with SIV_mac_239Δ*nef*, median 50% ADCC titers were 51-fold higher than those elicited by single-cycle SIV, and this difference expanded to 233-fold by week 21 (Figure 2C). The 50% ADCC titers (Figure 2C) and the AUC values for ADCC (Figure 2D) at any time point after inoculation with SIV_mac_239Δ*nef* were significantly higher than at either time point after inoculation with single-cycle SIV (2-tailed Mann-Whitney U tests, P = 0.0062 to P<0.0001). Thus, in contrast to persistent infection with SIV_mac_239Δ*nef*, repeated stimulation of antibody responses with SIV limited to a single cycle of infection did not elicit high ADCC titers.

ADCC titers measured after immunization with SIV_mac_239Δ*nef* and single-cycle SIV were compared with antibody titers that bind recombinant forms of SIV_mac_239 Env in enzyme-linked immunoadsorbent assays (ELISAs). Relationships among these measures of Env-specific antibody responses were evaluated by calculating Spearman correlation coefficients (R_S_). ELISA titers against SIV_mac_239 gp120 correlated with 50% ADCC titers against SIV_mac_239-infected cells (R_S_ = 0.8190, P<0.0001) (Figure 2E), and with AUC values for ADCC (R_S_ = 0.7809, P<0.0001) (Figure 2F). Although a linear relationship was observed between ADCC and gp120-binding titers in the animals persistently infected with SIV_mac_239Δ*nef*, plasma samples from the animals immunized with single-cycle SIV belonged to an out-group, which was displaced towards higher gp120-binding titers, relative to ADCC (Figure 2E and F). ELISA titers against gp140 also correlated with 50% ADCC titers (R_S_ = 0.9015, P<0.0001) (Figure 2G), and with AUC values for ADCC (R_S_ = 0.8836, P<0.0001) (Figure 2H). However, any displacement of the single-cycle SIV-immunized animals towards higher gp140-binding, relative to ADCC titers, was more subtle than that observed for gp120-binding titers (Figure 2E–H). This may reflect the occlusion of surfaces in gp140 oligomers that are exposed on gp120 monomers [39]. In comparison to persistent infection with SIV_mac_239Δ*nef*, immunization with single-cycle SIV may therefore stimulate a higher proportion of gp120-specific antibodies with low or undetectable ADCC activity against virus-infected cells, due to recognition of epitopes that are occluded in the native Env trimer.

### Recognition of heterologous Env proteins

**Figure 3:**
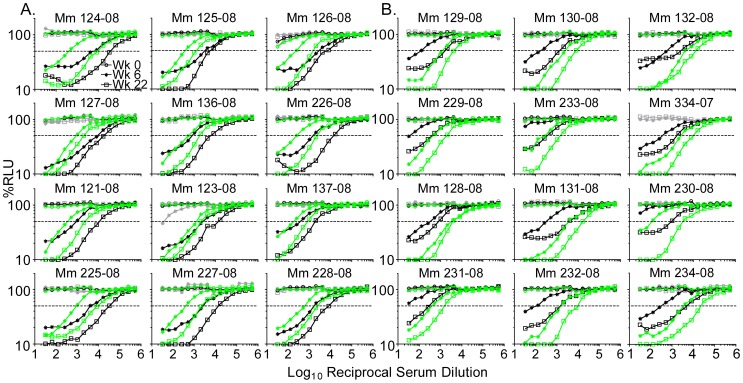
ADCC against viruses matched or mismatched to the vaccine strain in Env. Sera drawn 0, 6, or 22 weeks after inoculation with SIV_mac_239Δ*nef* (A) or with the recombinant vaccine strain SIV_mac_239Δ*nef*/E543-3*env* (B) were tested for ADCC against target cells infected with SIV_mac_239 (black), SIV_mac_239/E543-3*env* (green), or SHIV_SF162P3_ (gray). Dashed lines indicate 50% activity.

The ADCC activity against SIV strains that were matched or mismatched with the vaccine strain in Env was compared. Sera were collected from twelve macaques inoculated with SIV_mac_239Δ*nef* (Figure 3A), and twelve inoculated with a recombinant form of SIV_mac_239Δ*nef* containing the *env* gene of SIV_sm_E543-3 [40], designated SIV_mac_239Δ*nef*/E543-3*env* (Figure 3B). Sera from all 24 animals were tested for ADCC activity against target cells infected with SIV_mac_239 or SIV_mac_239/E543-3*env*. On average, the 50% ADCC titers were seven-fold higher when the vaccine and test viruses were matched in Env than when they were mismatched (2-tailed Wilcoxon matched pairs test, P<0.0001). The 50% ADCC titers were also approximately seven-fold higher at week 22 than at week 6 (2-tailed Wilcoxon matched pairs test, P<0.0001). Thus, the 50% ADCC titers against the Env-matched virus at week 6 and the Env-mismatched virus at week 22 were comparable. Therefore, ADCC titers against Env-mismatched viruses were lower and required more time to develop than ADCC titers against Env-matched viruses.

### ADCC activity is proportional to the extent of vaccine strain replication

**Figure 4:**
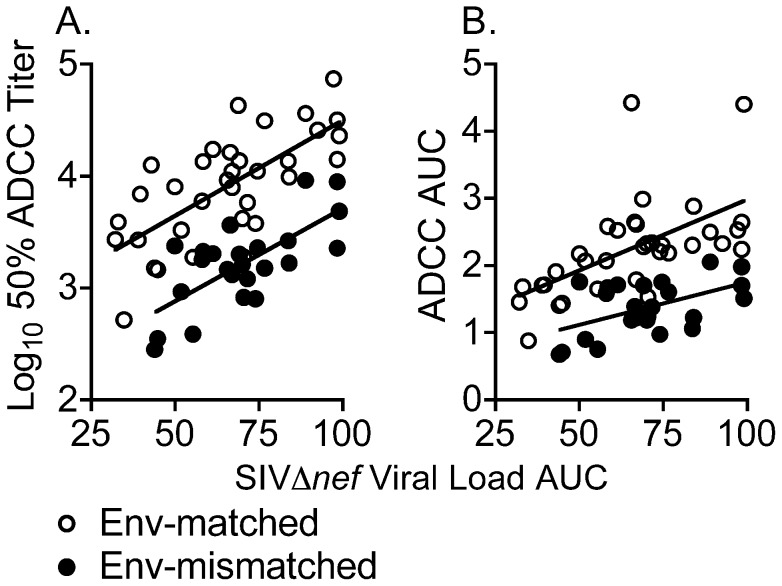
Relationship between the extent of vaccine strain replication and ADCC activity. The extent of SIV_mac_239Δ*nef* or SIV_mac_239Δ*nef*/E543-3*env* replication was estimated from the area under the curve (AUC) of log_10_-transformed vaccine strain viral loads over weeks 0 through 21 or 22, and compared to ADCC activity at week 21 or 22. Vaccine strain viral load AUC values were correlated with 50% ADCC titers (A) against Env-matched (R_S_ = 0.68, P<0.0001) and Env-mismatched (R_S_ = 0.55, P = 0.006) SIV strains, and also with AUC values for ADCC activity (B) against Env-matched (R_S_ = 0.64, P<0.0001) and Env-mismatched (R_S_ = 0.42, P = 0.0421) SIV strains. Linear regression lines are drawn.

The extent of vaccine strain replication was estimated by calculating AUC values for log_10_-transformed SIVΔ*nef* viral RNA loads in plasma over the first 21 or 22 weeks after inoculation. AUC values for viral loads among animals inoculated with SIV_mac_239Δ*nef* and SIV_mac_239Δ*nef*/E543-3*env* were similar, averaging 65 and 67 log_10_-transformed RNA copies per ml×weeks, respectively. The extent of vaccine strain replication by the end of this time period correlated with 50% ADCC titers against Env-matched (R_S_ = 0.68, P<0.0001) and Env-mismatched (R_S_ = 0.55, P = 0.006) viruses (Figure 4A), and also with AUC values for ADCC against Env-matched (R_S_ = 0.64, P<0.0001) and Env-mismatched (R_S_ = 0.42, P = 0.0421) viruses (Figure 4B). These relationships suggest that the development of antibodies that direct ADCC is driven by the extent of antigenic stimulation provided by vaccine strain replication.

### Higher ADCC among animals uninfected after SIV_mac_251 challenge

Twelve animals were challenged intravenously with SIV_mac_251_NE_ 46 weeks after inoculation with SIVΔ*nef*. The SIVΔ*nef* strain in six of these twelve animals was SIV_mac_239Δ*nef*, whereas the other six were inoculated with SIV_mac_239Δ*nef*/E543-3*env*. All twelve of these animals resisted two intravenous challenges with SIV_mac_239 on weeks 22 and 33, while three naïve control animals challenged on week 22 and two challenged on week 33 all became infected. When the twelve SIVΔ*nef*-immunized animals were subsequently re-challenged with SIV_mac_251_NE_ on week 46, three became infected, as did both naïve control animals challenged at the same time. Although all three immunized animals that became infected were among those inoculated with SIV_mac_239Δ*nef*/E543-3*env*, the trend toward more infections in this group was not significant (2-tailed Fisher's exact test, P = 0.18). Comparisons of SIVΔ*nef* viral loads for animals that became infected versus remained uninfected, or animals immunized with SIV_mac_239Δ*nef* versus SIV_mac_239Δ*nef*/E543-3*env*, did not reveal significant differences (Figure S1A–D). Likewise, sera collected on the day of challenge from the animals that remained uninfected by SIV_mac_251_NE_ did not have significantly higher binding titers against gp120 (2-tailed Mann-Whitney U test, P = 0.2091) (Figure S1E), or gp140 (2-tailed Mann-Whitney U test, P = 0.3727) (Figure S1F), in comparison to the animals that became infected.

**Figure 5:**
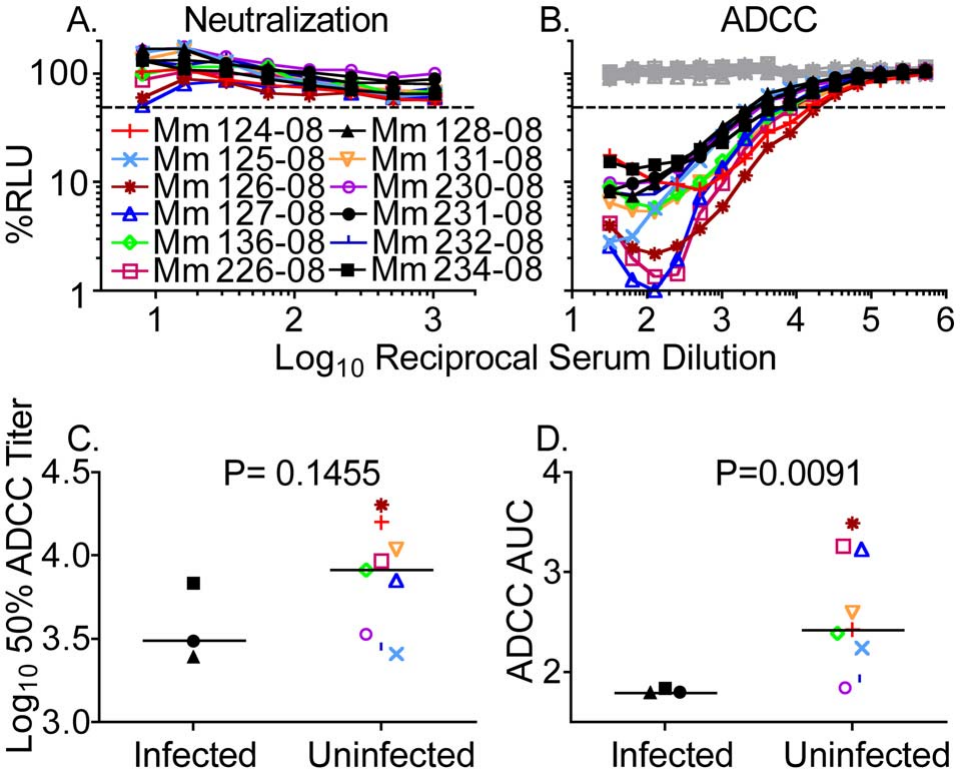
Neutralization and ADCC on the day of intravenous challenge with SIV_mac_251_NE_. Macaques were challenged with an intravenous dose of SIV_mac_251_NE_ on week 46 after inoculation with SIV_mac_239Δ*nef* or SIV_mac_239Δ*nef*/E543-3*env*. Sera collected the day of challenge were evaluated for neutralization of SIV_mac_251_NE_ (A) and ADCC against SIV_mac_251_NE_-infected cells (B). Solid black symbols indicate animals that became infected by SIV_mac_251_NE_. Dashed lines indicate 50% activity. Target cells infected with SHIV_SF162P3_ served as a negative control for ADCC assays (gray). Differences in 50% ADCC titers were not significant (C). However, AUC values for ADCC were higher among the immunized animals that remained uninfected versus the immunized animals that became infected (2-tailed Mann-Whitney U test, P = 0.0091) (D). None of these macaques had the MHC class I alleles *Mamu*-A*01, -B*08 or -B*17 associated with reduced viral replication [67]–[69].

Sera drawn the day of intravenous challenge with SIV_mac_251_NE_ were tested for neutralization of SIV_mac_251_NE_ and for ADCC against SIV_mac_251_NE_-infected cells. Neutralizing antibody titers were low to undetectable (Figure 5A), and differences among the infected versus uninfected animals were not significant at the highest serum concentration tested, a 1∶8 dilution (2-tailed Mann-Whitney U test, P = 0.3727). However, all of these samples had measureable ADCC activity (Figure 5B). Whereas differences in 50% ADCC titers were not significant (Figure 5C), the animals that remained uninfected by SIV_mac_251_NE_ had higher AUC values for ADCC than those that became infected (2-tailed Mann-Whitney U test, P = 0.0091) (Figure 5D). Thus, more complete elimination of the SIV_mac_251_NE_-infected target cells, as measured by AUC values for ADCC, was associated with protection against infection by intravenous challenge with SIV_mac_251_NE_.

To address the temporal association between the development of antibody responses and protective immunity, we studied animals that were challenged at different time points after inoculation with SIV_mac_239Δ*nef*. Groups of six female macaques were challenged by high-dose vaginal inoculation with SIV_mac_251_UCD_ at weeks five, twenty, or forty after immunization with SIV_mac_239Δ*nef* (Reeves et al., manuscript in preparation). All six animals challenged at week five became infected, as did three of six animals challenged at week twenty, and four of six animals challenged at week forty. Three naïve control animals challenged at each time point all became infected, except one animal challenged at week twenty. Peak SIV_mac_239Δ*nef* viral loads were unrelated to the outcome of challenge (Figure S2A). Although the total extent of vaccine strain replication, as estimated from AUC values for SIV_mac_239Δ*nef* viral loads, tended to be higher among the animals that remained uninfected compared to those that became infected, this trend was not significant (2-tailed Mann-Whitney U test, P = 0.0939) (Figure S2B). The animals that remained uninfected by SIV_mac_251_UCD_ also did not have significantly higher binding antibody titers than those that became infected, as measured by ELISA against gp120 (2-tailed Mann-Whitney U test, P = 0.4304) (Figure S2C) or gp140 (2-tailed Mann-Whitney U test, P = 0.1148) (Figure S2D).

**Figure 6:**
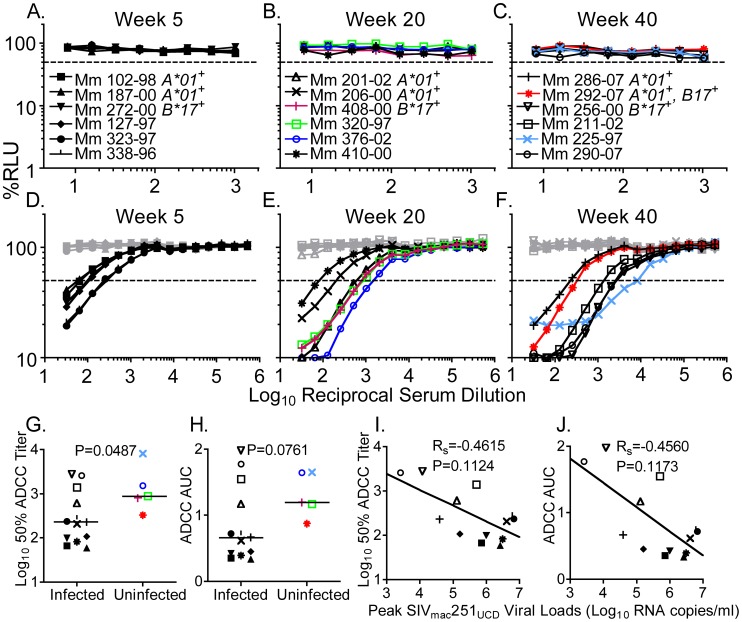
Neutralization and ADCC on the day of high-dose vaginal challenge with SIV_mac_251_UCD_. At 5, 20, or 40 weeks after inoculation with SIV_mac_239Δ*nef*, groups of 6 macaques each were challenged by high-dose vaginal inoculation with SIV_mac_251_UCD_. Serum collected the day of challenge was evaluated for neutralization of SIV_mac_251_UCD_ (A–C) and ADCC against SIV_mac_251_UCD_-infected cells (D–F). Symbols appear in color for immunized macaques that remained uninfected by SIV_mac_251_UCD_, and in black for the immunized animals that became infected. Target cells infected with SHIV_SF162P3_ served as a negative control for ADCC assays (gray). Dashed lines indicate 50% activity. The groups challenged on weeks 5, 20, and 40 were combined for statistical analysis (G–J). The SIV_mac_239Δ*nef*-immunized macaques remaining uninfected by SIV_mac_251_UCD_ had higher 50% ADCC titers than those that became infected (2-tailed Mann-Whitney U test, P = 0.0487) (G). A similar but non-significant trend was observed in AUC values for ADCC (2-tailed Mann-Whitney U test, P = 0.0761) (H). Non-significant trends were in the direction of lower peak SIV_mac_251_UCD_ viral loads for animals with higher 50% ADCC titers (R_S_ = −0.4615, P = 0.1124) (I) and higher AUC measurements of ADCC activity (R_S_ = −0.4560, P = 0.1173) (J). Linear regression lines are shown. The macaque with the lowest ADCC titers among those remaining uninfected was the only animal possessing the protective combination of MHC class I alleles Mamu-A*01 and -B*17 [70].

Sera collected on the day of challenge with SIV_mac_251_UCD_ were evaluated for neutralization of SIV_mac_251_UCD_ (Figure 6A–C). However, neutralization of SIV_mac_251_UCD_ was not detectable for any of these serum samples (Figure 6A–C). The absence of detectable neutralizing antibodies is a consequence of the inherent resistance of SIV_mac_251_UCD_ to neutralization (Figure S3). Indeed, SIV_mac_251_UCD_ is even more resistant to neutralization than SIV_mac_251_NE_ by plasma from animals chronically infected with SIV_mac_239 (2-tailed Wilcoxon matched pairs test, P = 0.0098) (Figure S3A), and by soluble CD4 (Figure S3B). The relative resistance of SIV_mac_251_UCD_ to antibody may have been a factor in the small number of animals that were protected against infection with this challenge strain.

The capacity of the same sera to direct ADCC against SIV_mac_251_UCD_-infected cells was evaluated (Figure 6D–F). In contrast to neutralization, all had measurable ADCC activity (Figure 6D–F). Statistically significant outcomes could not be reached at individual time points, or for a group of animals that combines just those challenged on weeks twenty and forty (Table S1). However, when the animals challenged five, twenty and forty weeks after inoculation with SIV_mac_239Δ*nef* were analyzed together, those that remained uninfected had higher 50% ADCC titers on the day they were challenged than those that became infected (2-tailed Mann-Whitney U test, P = 0.0487) (Figure 6G). A similar trend was observed for AUC values for ADCC, although these differences were not significant (Figure 6H). Also, among the animals that became infected, there was a trend towards higher ADCC activity in animals with lower peak SIV_mac_251_UCD_ viral loads (Figure 6I and J). Therefore, higher 50% ADCC titers present at later challenge time points after inoculation with SIV_mac_239Δ*nef* were associated with protection against infection by high-dose vaginal challenge with SIV_mac_251_UCD_.

## Discussion

Identifying the immune responses that mediate protection by live-attenuated SIV and understanding their induction may inspire strategies for engineering a safe and effective vaccine against HIV-1. We hypothesized that antibody functions other than neutralization contribute to the protective immunity provided by live-attenuated SIV against wild-type pathogenic SIV challenge. Here we demonstrate that properties of the antibody response reflected in ADCC titers mirror hallmarks of protection by live-attenuated SIV. The protective immunity conferred by live-attenuated SIV increases over time [7], [8], is usually incomplete against heterologous challenge [41], [42], and is greater for vaccine strains that replicate at higher levels [7], [9]. In accordance with these observations, our data indicate that ADCC titers increase progressively over time, are lower against viruses expressing heterologous Env proteins, and are proportional to the extent of vaccine strain replication. Furthermore, in two different challenge experiments, measures of ADCC activity were associated with protection against infection by SIV_mac_251.

In one experiment, macaques inoculated with SIV_mac_239Δ*nef* or SIV_mac_239Δ*nef*/E543-3*env* that remained uninfected after intravenous challenge with SIV_mac_251_NE_ had higher AUC values for ADCC than those that became infected. In another experiment, animals that remained uninfected after high-dose vaginal challenge with SIV_mac_251_UCD_ at different time points after inoculation with SIV_mac_239Δ*nef* had higher 50% ADCC titers than those that became infected. Differences in AUC values for ADCC were significant in one experiment, whereas differences in 50% ADCC titers were significant in the other, perhaps reflecting the limited power to detect differences using small numbers of infected versus uninfected animals. Additional differences between the two studies, including the greater resistance of SIV_mac_251_UCD_ than SIV_mac_251_NE_ to antibodies, the greater length of time allowed for the maturation of antibody responses prior to challenge with SIV_mac_251_NE_ (46 weeks) than SIV_mac_251_UCD_ (5, 20, and 40 weeks), and the effect of mismatches in Env between SIV_mac_239Δ*nef*/E543-3*env* and SIV_mac_251_NE_, may also have contributed to the detection of differences in AUC values for ADCC against SIV_mac_251_NE_. Although differences between the two SIV_mac_251 challenge experiments may have favored one method of data analysis over the other, measures of ADCC activity were associated with complete protection in both experiments.

While the relationship between ADCC activity and the outcome of challenge suggests that these antibodies contribute to protection, correlation does not establish causation. In addition to ADCC, Fc receptor crosslinking stimulates the secretion of molecules that promote lymphocyte homing and activation, and that may inhibit virus replication [26], [27]. The antibodies that direct ADCC may also mediate effector functions through complement fixation [25], [28]. Furthermore, ADCC assays may measure antibodies that block virus infection at concentrations present *in vivo*, but are undetectable using conventional neutralization assays. Therefore, although ADCC may be an important effector mechanism [43], ADCC could also be a surrogate for other effector mechanisms that contribute to protection. Mechanisms of immunity not mediated by antibodies may also covary with ADCC activity. For instance, T cell, antibody, and innate immune responses may all be affected by the extent of antigenic stimulation. It is conceivable that the observed relationships are due to differences that exist among animals inoculated with SIV_mac_239Δ*nef* versus SIV_mac_239Δ*nef*/E543-3*env*, or among animals challenged five, twenty, and forty weeks after inoculation with SIV_mac_239Δ*nef*. Thus, while our findings implicate antibodies in protection by live-attenuated SIV, they do not preclude a role for other immune responses.

More than one type of immune response elicited by live-attenuated SIV may be necessary for complete protection against SIV_mac_251 challenge. Passive transfer experiments in different live-attenuated SIV vaccine models have yielded mixed results regarding the ability of antibodies alone to protect against SIV infection, demonstrating complete protection in one study [44], and no protection in another [45]. In contrast to the absence of detectable neutralizing antibodies in the completely protected animals in this study, relatively high concentrations of neutralizing monoclonal antibodies were necessary to protect macaques against SHIV infection in passive transfer experiments [43], [46]–[48]. T cell responses present in macaques inoculated with SIVΔ*nef* [14]–[16], but absent in macaques that received antibodies passively, may help to explain these differences in neutralizing antibody titers required for complete protection.

Our observations are in agreement with other reports that have associated antibody responses with protection. The Robert-Guroff laboratory, and others, have associated lower viral loads after infection with higher ADCC activity measured using target cells that were coated with monomeric gp120 [32], [36], [37], recombinant gp140 [33], or infected with T cell line-adapted SIV [49]. However, in these studies, ADCC was not associated with protection from infection, or measured using target cells infected with neutralization-resistant viruses. Nevertheless, antibodies that bound recombinant forms of gp120 by ELISA and that neutralized neutralization-sensitive SIV strains were associated with a reduced rate of infection [37]. Consistent with these observations on vaccine protection, a recent study on mother-to-child transmission of HIV-1 found that the breast milk of mothers whose newborns remained uninfected contained antibodies with higher ADCC activity against gp120-coated target cells [50]. In the context of vaccination with different live-attenuated strains of SIV, antibody avidity was also associated with resistance to infection and lower post-challenge viral loads after vaginal challenge with SIV_mac_251_NE_ [9]. Likewise, neutralization of SIV_mac_251_NE_ at a 1∶4 dilution of serum was associated with protection in a combined group of animals that remained uninfected or strongly controlled SIV_mac_251_NE_ viral loads [7]. Taken together, these studies support a role for antibodies in protective immunity.

Interest in antibody functions other than neutralization has recently increased as a result of the RV144 trial, in which a modest reduction in the rate of HIV-1 infection was reported among recipients of a recombinant canarypox vector prime and gp120 protein boost vaccine [51]. Virus-specific CD8^+^ T cell responses were not measurably different between vaccinated and unvaccinated trial participants. Whereas antibodies capable of neutralizing primary HIV-1 isolates were also undetectable among vaccinated individuals, gp120-binding titers were consistently detectable by ELISA. Functions of antibodies other than neutralization have therefore been postulated to potentially be responsible for protection in the RV144 trial [52]. Among six primary variables in the immune correlates analysis of the RV144 trial, IgG titers to the V2 region of gp120 were associated with protection, whereas Env-specific IgA antibodies were associated with a higher risk of infection [53]. There was also a non-significant trend towards a lower risk of HIV-1 infection among vaccine recipients with higher ADCC activity using the assay described here. This relationship reached borderline statistical significance after excluding subjects with Env-specific IgA in plasma [53]. These observations further support a role for antibodies in vaccine protection against immunodeficiency virus infection.

Persistent expression of Env may be essential to elicit protective antibody responses. The progressive increases in ADCC activity over time, and the considerably higher ADCC activity elicited by SIV_mac_239Δ*nef* versus single-cycle SIV, imply that the persistent antigenic stimulation provided by ongoing SIV_mac_239Δ*nef* replication is important for the development of high ADCC titers. Differences in the maturation of antibody responses may also contribute to the better protection provided by SIVΔ*nef* in comparison to single-cycle SIV [38]. Furthermore, a longer period of persistent infection with SIVΔ*nef* was required for ADCC titers against SIV strains expressing heterologous Env proteins to reach the levels observed at an earlier time point against the Env-matched strain. Persistent Env expression may therefore be required to elicit antibodies with high and broadly reactive ADCC activity against circulating HIV-1 strains with diverse neutralization-resistant Env proteins.

A vaccine against HIV-1 must contend with a degree of sequence variation that typically renders neutralizing sera ineffective against heterologous HIV-1 strains isolated from other people [19], [54]. The Env proteins of SIV_mac_239 and SIV_sm_E543-3 differ in amino acid sequence by 18%, which approximates the median difference between the Env proteins of individual HIV-1 isolates within a clade [54], [55]. Therefore, the ADCC titers observed against cells infected with neutralization-resistant Env-mismatched target viruses suggest that antibodies may have broader efficacy against heterologous HIV-1 isolates than is generally revealed by neutralization assays.

In summary, we show that properties of the antibody response elicited by SIVΔ*nef* mirror hallmarks of protection by live-attenuated SIV, and that ADCC activity is associated with apparent sterilizing protection against SIV_mac_251. These observations support a role for antibodies in protection by live-attenuated SIV, despite the paradoxical absence of detectable neutralizing antibody titers against the challenge virus in most fully protected animals. The temporal analyses of ADCC activity against both Env-matched and Env-mismatched viruses, and the significantly higher ADCC titers observed in SIVΔ*nef*-infected animals than in animals repeatedly immunized with single-cycle SIV, suggest that persistent Env expression may be necessary to drive the maturation of high-titer, broadly reactive antibody responses. Therefore, strategies designed to persistently stimulate Env-specific antibodies may significantly improve the efficacy of vaccines against HIV-1.

## Materials and Methods

### ADCC assay

Due to the variability and limited scalability of assays dependent upon primary cells, we engineered a pair of cell lines to serve as targets and effectors in ADCC assays. The target cells were derived from CEM.NKR-CCR5 CD4^+^ T cells [56], [57] (AIDS Research and Reference Reagent Program, Division of AIDS, NIAID, NIH, contributed by Dr. Alexandra Trkola). These were transduced with a pLNSX-derived retroviral vector to express firefly luciferase under the transcriptional regulation of the SIV LTR promoter [23]. Target cells were maintained in “R10” cell culture media consisting of RPMI (Invitrogen) supplemented with 10% fetal bovine serum (FBS) (Invitrogen), 25 mM HEPES (Invitrogen), 2 mM L-glutamine (Invitrogen), and 0.1 mg/ml Primocin (InvivoGen). Effector cells were derived from the CD16-negative human NK cell line KHYG-1 [58] (Japan Health Sciences Foundation) by stable transduction with a pQCXIN-derived retroviral vector expressing rhesus macaque CD16 (FCGR3A variant 7) [59]. KHYG-1 effector cells were maintained at a density of 1×10^5^ to 4×10^5^ cells per ml in R10 media supplemented with IL-2 at 10 U per ml (Roche) and cyclosporine A at 1 µg per ml (Sigma).

Four days prior to each ADCC assay, the target cells were infected by spinoculation [60]. On the day of the assay, infected target cells were washed three times with R10 immediately before use. ADCC assays were conducted in round-bottom 96-well plates. Each well contained 10^5^ effector cells and 10^4^ target cells. Effector and target cells were incubated together for eight hours in the presence of triplicate serial two-fold dilutions of serum or plasma before the luciferase activity was measured using the luciferase substrate reagent BriteLite Plus (Perkin Elmer). Relative light units (RLU) indicate luciferase expression by infected target cells. Wells containing uninfected target cells plus effector cells defined 0% RLU, and wells containing infected target cells plus effector cells with no serum or plasma defined 100% RLU. The %ADCC activity was defined as 100 minus %RLU.

Antibodies from some macaques bound uninfected CEM.NKR-CCR5 cells. To deplete these antibodies, 10^7^ uninfected target cells were resuspended in the sample and incubated for twenty minutes at room temperature. This process was repeated twenty times for the animals immunized with single-cycle SIV, and twelve times for the animals in the Env-mismatch and vaginal challenge studies.

### Neutralization assays

Neutralization was measured as previously described [23], [61]. The sensitivity of the virus neutralization assay was maximized by minimizing the amount of virus input required to obtain a consistent level of infected C8166-secreted alkaline phosphatase (SEAP) reporter cells. These amounts were 0.5 ng p27 SIV_mac_239, 5 ng p27 SIV_mac_251_NE_, and 0.5 ng p27 SIV_mac_251_UCD_ per well. However, the resistance of SIV_mac_251_NE_, SIV_mac_251_UCD_, and SIV_mac_251_TCLA_ to neutralization by sCD4-IgG was evaluated using 5 ng p27 per well for all 3 viruses. Each well contained 15,000 C8166-SEAP cells. Plasma or serum dilutions were pre-incubated with virus for one hour at 37°C before adding C8166-SEAP cells. After three days, SEAP activity was determined using a luminescent assay (Applied Biosystems).

### ELISAs

Recombinant 6-His tagged SIV_mac_239 gp120 or gp140 protein (Immune Technology) diluted to 0.5 µg/ml in 0.1 M sodium bicarbonate pH 9.5 was coated onto Maxisorb ELISA plates (NUNC). Plates were blocked using PBS containing 0.5% TWEEN-20 (Sigma) and 5% blotting-grade non-fat dry milk (NFDM) blocker (BioRad). Antibody dilutions were made in PBS containing 0.5% TWEEN-20 and 5% NFDM. Bound IgG was detected using a horseradish peroxidase conjugated goat anti-monkey/human IgG antibody (Santa Cruz Biotechnology). The upper limit of a 95% confidence interval calculated using plasma from 10 naïve macaques diluted 1∶100 served as the endpoint titer [62].

### Animals

The animals were Indian-origin rhesus macaques (*Macaca mulatta*) housed in a biocontainment facility at the New England Primate Research Center (NEPRC), and given care in accordance with standards of the Association for Assessment and Accreditation of Laboratory Animal Care and the Harvard Medical School Animal Care and Use Committee. The animal samples used here were collected under experimental protocols approved by the Harvard Medical Area Standing Committee on Animals, and conducted in accordance to the *Guide for the Care and Use of Laboratory Animals* [63]. Additional analyses using these animals will be published separately by R. C. Desrosiers and by R. K. Reeves.

### Viruses

The intravenous SIV_mac_239 challenge dose used on week 22 consisted of twenty animal-infectious doses of virus produced by transfection of 293T cells. The week 33 intravenous challenge with SIV_mac_239 contained ten animal-infectious doses of a rhesus PBMC-derived virus stock used previously [38]. The intravenous SIV_mac_251_NE_ challenge was ten animal-infectious doses (32 pg p27) of a rhesus PBMC stock prepared in February of 1991, used in other studies [7]–[9]. Vaginal challenges consisted of two inoculations on one day of 1 ml undiluted SIV_mac_251_UCD_ [64] (100 ng p27), prepared at the California National Primate Research Center in June of 2004. Neutralization and ADCC assays were done using SIV_mac_239 and SIV_mac_239/E543-3*env* produced by transfection of 293T cells, and SIV_mac_251_NE_ and SIV_mac_251_UCD_ expanded from the corresponding uncloned challenge stocks in rhesus macaque PBMC. SHIV_SF162P3_ was also expanded in rhesus PBMC (AIDS Research and Reference Reagent Program, NIAID, NIH, contributed by Drs. Janet Harouse, Cecilia Cheng-Mayer, Ranajit Pal and the DAIDS, NIAID). The rhesus PBMC used to expand virus stocks were depleted of CD8^+^ cells using Dynal anti-CD8 magnetic beads (Invitrogen), activated with phytohemagglutinin (PHA) (Sigma), and then cultured in IL-2 (Roche). SIV_mac_251_TCLA_ was grown in MT4 cells.

### Plasma viral RNA load measurements

Challenge viruses were detected using primers specific for the *nef* sequences of SIV_mac_239 or SIV_mac_251 within the deletion in SIV_mac_239Δ*nef* [65]. The primers specific for wild-type SIV_mac_239 were GAATACTCCATGGAGAAACCCAGC and ATTGCCAATTTGTAACTCATTGTTCTTAG, and the labeled probe had the sequence CTTTTGGCCTCACTGATACCCCTAC. To reflect the polymorphic nature of the uncloned SIV_mac_251 virus stocks, the primer set designed to amplify SIV_mac_251 contained degenerate bases P and K, which mimic mixtures of C and T or A and G, respectively (GlenResearch). The SIV_mac_251-specific primers were GAATACPCCATGGAKAAACCCAGC and TGCCAATTTGTAA(C,T,G)TCATTGPTCTTAGG, and the SIV_mac_251-specific probe sequence was TAGAPGAGGAAGATGATGACTTGKTAGGG. Complete or apparent sterilizing protection was defined as the absence of detectable wild-type viral RNA from plasma at every post-challenge time point using the above primer/probe sets in a real-time RT-PCR assay with a nominal threshold of detection of 10–30 copies of RNA per ml [66].

### Statistical analysis

Fifty percent titers were calculated as the dilution at which a line connecting the values above and below 50% RLU would intercept the 50% RLU line. AUC values for ADCC were calculated such that they would be proportional to 50% ADCC titers, and represent the areas between 100% RLU and the titration curves as they appear in the figures. Whereas %ADCC, defined as 100% minus %RLU, appears asymptotic as it approaches 100%, minimum %RLU values are inversely proportional to 50% ADCC titers. Therefore, AUC values for ADCC were calculated from values for log_10_100 minus log_10_%RLU, which were summed over all dilutions. This sum was multiplied by the log_10_-transformed dilution factor of two to find an area. The ability of ADCC activity to predict neutralization was evaluated by logistic regression using SPSS (IBM). The statistical significance of other comparisons was evaluated in Prism version 4.1b (GraphPad Software) using 2-tailed Mann-Whitney U tests, 2-tailed Fisher's exact tests, 2-tailed Wilcoxon matched pairs tests, and Spearman correlation coefficients.

## Supporting Information

Figure S1 **SIVΔ** ***nef*** **viral loads and ELISA titers among animals challenged with SIV_mac_251_NE_.** There were no significant differences in vaccine strain viral loads among the animals that became infected versus those that remained uninfected after intravenous challenge with SIV_mac_251_NE_ in terms of peak log_10_ RNA copies per ml (2-tailed Mann-Whitney U test, P = 0.8636) (A), or AUC log_10_ RNA copies per ml×weeks for the period of weeks 0–46 after inoculation (2-tailed Mann-Whitney U test, P = 0.2091) (B). SIV_mac_239Δ*nef* and SIV_mac_239Δ*nef*/E543-3*env* did not differ significantly in peak vaccine strain viral loads (2-tailed Mann-Whitney U test, P = 0.8182) (C), or in AUC values for vaccine strain viral loads over weeks 0–46 (P = 0.9372) (D). The outcome of challenge was not significantly related to binding antibodies measured by ELISA against recombinant SIV_mac_239 gp120 (2-tailed Mann-Whitney U test, P = 0.2091) (E), or gp140 (2-tailed Mann-Whitney U test, P = 0.3727) (F).
(TIFF)

Figure S2 **SIV_mac_239Δ** ***nef*** **viral loads and ELISA titers among animals challenged with SIV_mac_251_UCD_.** Peak log_10_ SIV_mac_239Δ*nef* viral loads appeared unrelated to protection against infection by SIV_mac_251_UCD_ (2-tailed Mann-Whitney U test, P = 0.8437) (A). SIV_mac_239Δ*nef* AUC log_10_ RNA copies per ml×weeks for the period through the day of challenge with SIV_mac_251_UCD_ at 5, 20, or 40 weeks after inoculation appeared higher among the animals that remained uninfected, but this difference was not significant (2-tailed Mann-Whitney U test, P = 0.0939) (B). The SIV_mac_239Δ*nef*-immunized animals that remained uninfected did not have significantly higher ELISA titers in sera collected on the day of challenge than those that became infected against SIV_mac_239 gp120 (2-tailed Mann-Whitney U test, P = 0.4304) (C), or gp140 (2-tailed Mann-Whitney U test, P = 0.1148) (D).
(TIFF)

Figure S3 **Neutralization resistance of SIV_mac_251 strains.** SIV_mac_251_NE_, SIV_mac_251_UCD_, and T cell line-adapted SIV_mac_251_TCLA_ were compared for their relative resistance to neutralization. Plasma samples from 16 macaques chronically infected with SIV_mac_239 were titered for neutralization of each strain (A). SIV_mac_251_NE_ and SIV_mac_251_UCD_ are both more resistant to neutralization by plasma than SIV_mac_251_TCLA_ (2-tailed Wilcoxon matched pairs tests, P = 0.0006). SIV_mac_251_UCD_ is more resistant to neutralization by plasma than SIV_mac_251_NE_ (2-tailed Wilcoxon matched pairs test, P = 0.0098). SIV_mac_251_NE_, SIV_mac_251_UCD_, SIV_mac_251_TCLA_ were also compared for their sensitivity to neutralization by a soluble CD4 protein, sCD4-IgG, which consists of human CD4 domains 1 and 2 fused to the IgG1 heavy chain (B). The dashed line indicates 50% of maximal infectivity.
(TIFF)

Table S1 **Significance of differences among animals infected versus uninfected after SIV_mac_251_UCD_ challenge.** Differences among infected versus uninfected animals in peak SIV_mac_239Δ*nef* viral loads, total SIV_mac_239Δ*nef* replication (estimated from AUC values for log_10_ RNA copies per ml×weeks), gp120 ELISA titers, gp140 ELISA titers, 50% ADCC titers, and AUC values for ADCC were evaluated for significance by 2-tailed Mann-Whitney U tests. Significance was not determined (ND) for comparisons that included less than 3 animals in one group.
(DOCX)

## References

[1] Alexander L, Illyinskii PO, Lang SM, Means RE, Lifson J, et al (2003) Determinants of increased replicative capacity of serially passaged simian immunodeficiency virus with nef deleted in rhesus monkeys. J Virol 77: 6823–6835. 12768002 10.1128/JVI.77.12.6823-6835.2003 PMC156171

[2] Hofmann-Lehmann R, Vlasak J, Williams AL, Chenine AL, McClure HM, et al (2003) Live attenuated, nef-deleted SIV is pathogenic in most adult macaques after prolonged observation. AIDS 17: 157–166. 12545074 10.1097/00002030-200301240-00004

[3] Baba TW, Jeong YS, Pennick D, Bronson R, Greene MF, et al (1995) Pathogenicity of live, attenuated SIV after mucosal infection of neonatal macaques. Science 267: 1820–1825. 7892606 10.1126/science.7892606

[4] Baba TW, Liska V, Khimani AH, Ray NB, Dailey PJ, et al (1999) Live attenuated, multiply deleted simian immunodeficiency virus causes AIDS in infant and adult macaques. Nat Med 5: 194–203. 9930868 10.1038/5557

[5] Wyand MS, Manson KH, Lackner AA, Desrosiers RC (1997) Resistance of neonatal monkeys to live attenuated vaccine strains of simian immunodeficiency virus. Nat Med 3: 32–36. 8986737 10.1038/nm0197-32

[6] Daniel MD, Kirchhoff F, Czajak SC, Sehgal PK, Desrosiers RC (1992) Protective effects of a live attenuated SIV vaccine with a deletion in the nef gene. Science 258: 1938–1941. 1470917 10.1126/science.1470917

[7] Wyand MS, Manson KH, Garcia-Moll M, Montefiori D, Desrosiers RC (1996) Vaccine protection by a triple deletion mutant of simian immunodeficiency virus. J Virol 70: 3724–3733. 8648707 10.1128/jvi.70.6.3724-3733.1996 PMC190248

[8] Connor RI, Montefiori DC, Binley JM, Moore JP, Bonhoeffer S, et al (1998) Temporal analyses of virus replication, immune responses, and efficacy in rhesus macaques immunized with a live, attenuated simian immunodeficiency virus vaccine. J Virol 72: 7501–7509. 9696847 10.1128/jvi.72.9.7501-7509.1998 PMC109989

[9] Johnson RP, Lifson JD, Czajak SC, Cole KS, Manson KH, et al (1999) Highly attenuated vaccine strains of simian immunodeficiency virus protect against vaginal challenge: inverse relationship of degree of protection with level of attenuation. J Virol 73: 4952–4961. 10233957 10.1128/jvi.73.6.4952-4961.1999 PMC112539

[10] Koff WC, Johnson PR, Watkins DI, Burton DR, Lifson JD, et al (2006) HIV vaccine design: insights from live attenuated SIV vaccines. Nat Immunol 7: 19–23. 16357854 10.1038/ni1296

[11] Carroll MC (1998) The role of complement and complement receptors in induction and regulation of immunity. Annu Rev Immunol 16: 545–568. 9597141 10.1146/annurev.immunol.16.1.545

[12] Dittmer U, Brooks DM, Hasenkrug KJ (1999) Requirement for multiple lymphocyte subsets in protection by a live attenuated vaccine against retroviral infection. Nat Med 5: 189–193. 9930867 10.1038/5550

[13] Nimmerjahn F, Ravetch JV (2008) Fcgamma receptors as regulators of immune responses. Nat Rev Immunol 8: 34–47. 18064051 10.1038/nri2206

[14] Johnson RP, Glickman RL, Yang JQ, Kaur A, Dion JT, et al (1997) Induction of vigorous cytotoxic T-lymphocyte responses by live attenuated simian immunodeficiency virus. J Virol 71: 7711–7718. 9311855 10.1128/jvi.71.10.7711-7718.1997 PMC192122

[15] Gauduin MC, Glickman RL, Ahmad S, Yilma T, Johnson RP (1999) Immunization with live attenuated simian immunodeficiency virus induces strong type 1 T helper responses and beta-chemokine production. Proc Natl Acad Sci U S A 96: 14031–14036. 10570193 10.1073/pnas.96.24.14031 PMC24185

[16] Gauduin MC, Yu Y, Barabasz A, Carville A, Piatak M, et al (2006) Induction of a virus-specific effector-memory CD4+ T cell response by attenuated SIV infection. J Exp Med 203: 2661–2672. 17116733 10.1084/jem.20060134 PMC2118155

[17] Sharpe SA, Cope A, Dowall S, Berry N, Ham C, et al (2004) Macaques infected long-term with attenuated simian immunodeficiency virus (SIVmac) remain resistant to wild-type challenge, despite declining cytotoxic T lymphocyte responses to an immunodominant epitope. J Gen Virol 85: 2591–2602. 15302953 10.1099/vir.0.80050-0

[18] Berek C, Berger A, Apel M (1991) Maturation of the immune response in germinal centers. Cell 67: 1121–1129. 1760840 10.1016/0092-8674(91)90289-b

[19] Richman DD, Wrin T, Little SJ, Petropoulos CJ (2003) Rapid evolution of the neutralizing antibody response to HIV type 1 infection. Proc Natl Acad Sci U S A 100: 4144–4149. 12644702 10.1073/pnas.0630530100 PMC153062

[20] Scheid JF, Mouquet H, Ueberheide B, Diskin R, Klein F, et al (2011) Sequence and structural convergence of broad and potent HIV antibodies that mimic CD4 binding. Science 333: 1633–1637. 21764753 10.1126/science.1207227 PMC3351836

[21] Wu X, Zhou T, Zhu J, Zhang B, Georgiev I, et al (2011) Focused evolution of HIV-1 neutralizing antibodies revealed by structures and deep sequencing. Science 333: 1593–1602. 21835983 10.1126/science.1207532 PMC3516815

[22] Cole KS, Rowles JL, Jagerski BA, Murphey-Corb M, Unangst T, et al (1997) Evolution of envelope-specific antibody responses in monkeys experimentally infected or immunized with simian immunodeficiency virus and its association with the development of protective immunity. J Virol 71: 5069–5079. 9188572 10.1128/jvi.71.7.5069-5079.1997 PMC191740

[23] Means RE, Greenough T, Desrosiers RC (1997) Neutralization sensitivity of cell culture-passaged simian immunodeficiency virus. J Virol 71: 7895–7902. 9311879 10.1128/jvi.71.10.7895-7902.1997 PMC192146

[24] Rook AH, Lane HC, Folks T, McCoy S, Alter H, et al (1987) Sera from HTLV-III/LAV antibody-positive individuals mediate antibody-dependent cellular cytotoxicity against HTLV-III/LAV-infected T cells. J Immunol 138: 1064–1067. 3027168

[25] Spear GT, Sullivan BL, Landay AL, Lint TF (1990) Neutralization of human immunodeficiency virus type 1 by complement occurs by viral lysis. J Virol 64: 5869–5873. 1700828 10.1128/jvi.64.12.5869-5873.1990 PMC248749

[26] Guidotti LG, Chisari FV (2001) Noncytolytic control of viral infections by the innate and adaptive immune response. Annu Rev Immunol 19: 65–91. 11244031 10.1146/annurev.immunol.19.1.65

[27] Forthal DN, Landucci G, Daar ES (2001) Antibody from patients with acute human immunodeficiency virus (HIV) infection inhibits primary strains of HIV type 1 in the presence of natural-killer effector cells. J Virol 75: 6953–6961. 11435575 10.1128/JVI.75.15.6953-6961.2001 PMC114423

[28] Guo RF, Ward PA (2005) Role of C5a in inflammatory responses. Annu Rev Immunol 23: 821–852. 15771587 10.1146/annurev.immunol.23.021704.115835

[29] Ackerman ME, Moldt B, Wyatt RT, Dugast AS, McAndrew E, et al (2011) A robust, high-throughput assay to determine the phagocytic activity of clinical antibody samples. J Immunol Methods 366: 8–19. 21192942 10.1016/j.jim.2010.12.016 PMC3050993

[30] Gomez-Roman VR, Florese RH, Patterson LJ, Peng B, Venzon D, et al (2006) A simplified method for the rapid fluorometric assessment of antibody-dependent cell-mediated cytotoxicity. J Immunol Methods 308: 53–67. 16343526 10.1016/j.jim.2005.09.018

[31] Chung AW, Rollman E, Center RJ, Kent SJ, Stratov I (2009) Rapid degranulation of NK cells following activation by HIV-specific antibodies. J Immunol 182: 1202–1210. 19124764 10.4049/jimmunol.182.2.1202

[32] Hidajat R, Xiao P, Zhou Q, Venzon D, Summers LE, et al (2009) Correlation of vaccine-elicited systemic and mucosal nonneutralizing antibody activities with reduced acute viremia following intrarectal simian immunodeficiency virus SIVmac251 challenge of rhesus macaques. J Virol 83: 791–801. 18971271 10.1128/JVI.01672-08 PMC2612365

[33] Xiao P, Zhao J, Patterson LJ, Brocca-Cofano E, Venzon D, et al (2010) Multiple vaccine-elicited nonneutralizing antienvelope antibody activities contribute to protective efficacy by reducing both acute and chronic viremia following simian/human immunodeficiency virus SHIV89.6P challenge in rhesus macaques. J Virol 84: 7161–7173. 20444898 10.1128/JVI.00410-10 PMC2898229

[34] Chung AW, Isitman G, Navis M, Kramski M, Center RJ, et al (2011) Immune escape from HIV-specific antibody-dependent cellular cytotoxicity (ADCC) pressure. Proc Natl Acad Sci U S A 108: 7505–7510. 21502492 10.1073/pnas.1016048108 PMC3088575

[35] Isitman G, Chung AW, Navis M, Kent SJ, Stratov I (2011) Pol as a target for antibody dependent cellular cytotoxicity responses in HIV-1 infection. Virology 412: 110–116. 21269655 10.1016/j.virol.2010.12.044 PMC3056898

[36] Ferrari G, Pollara J, Kozink D, Harms T, Drinker M, et al (2011) An HIV-1 gp120 envelope human monoclonal antibody that recognizes a C1 conformational epitope mediates potent antibody-dependent cellular cytotoxicity (ADCC) activity and defines a common ADCC epitope in human HIV-1 serum. J Virol 85: 7029–7036. 21543485 10.1128/JVI.00171-11 PMC3126567

[37] Barouch DH, Liu J, Li H, Maxfield LF, Abbink P, et al (2012) Vaccine protection against acquisition of neutralization-resistant SIV challenges in rhesus monkeys. Nature 482: 89–93. 22217938 10.1038/nature10766 PMC3271177

[38] Jia B, Ng SK, DeGottardi MQ, Piatak M, Yuste E, et al (2009) Immunization with single-cycle SIV significantly reduces viral loads after an intravenous challenge with SIV(mac)239. PLoS Pathog 5: e1000272. 19165322 10.1371/journal.ppat.1000272 PMC2621341

[39] Wyatt R, Desjardin E, Olshevsky U, Nixon C, Binley J, et al (1997) Analysis of the interaction of the human immunodeficiency virus type 1 gp120 envelope glycoprotein with the gp41 transmembrane glycoprotein. J Virol 71: 9722–9731. 9371638 10.1128/jvi.71.12.9722-9731.1997 PMC230282

[40] Hirsch V, Adger-Johnson D, Campbell B, Goldstein S, Brown C, et al (1997) A molecularly cloned, pathogenic, neutralization-resistant simian immunodeficiency virus, SIVsmE543-3. J Virol 71: 1608–1620. 8995688 10.1128/jvi.71.2.1608-1620.1997 PMC191219

[41] Wyand MS, Manson K, Montefiori DC, Lifson JD, Johnson RP, et al (1999) Protection by live, attenuated simian immunodeficiency virus against heterologous challenge. J Virol 73: 8356–8363. 10482586 10.1128/jvi.73.10.8356-8363.1999 PMC112853

[42] Reynolds MR, Weiler AM, Weisgrau KL, Piaskowski SM, Furlott JR, et al (2008) Macaques vaccinated with live-attenuated SIV control replication of heterologous virus. J Exp Med 205: 2537–2550. 18838548 10.1084/jem.20081524 PMC2571929

[43] Hessell AJ, Hangartner L, Hunter M, Havenith CE, Beurskens FJ, et al (2007) Fc receptor but not complement binding is important in antibody protection against HIV. Nature 449: 101–104. 17805298 10.1038/nature06106

[44] Van Rompay KK, Berardi CJ, Dillard-Telm S, Tarara RP, Canfield DR, et al (1998) Passive immunization of newborn rhesus macaques prevents oral simian immunodeficiency virus infection. J Infect Dis 177: 1247–1259. 9593009 10.1086/515270

[45] Almond N, Rose J, Sangster R, Silvera P, Stebbings R, et al (1997) Mechanisms of protection induced by attenuated simian immunodeficiency virus. I. Protection cannot be transferred with immune serum. J Gen Virol 78: 1919–1922. 9266988 10.1099/0022-1317-78-8-1919

[46] Parren PW, Marx PA, Hessell AJ, Luckay A, Harouse J, et al (2001) Antibody protects macaques against vaginal challenge with a pathogenic R5 simian/human immunodeficiency virus at serum levels giving complete neutralization in vitro. J Virol 75: 8340–8347. 11483779 10.1128/JVI.75.17.8340-8347.2001 PMC115078

[47] Hessell AJ, Rakasz EG, Poignard P, Hangartner L, Landucci G, et al (2009) Broadly neutralizing human anti-HIV antibody 2G12 is effective in protection against mucosal SHIV challenge even at low serum neutralizing titers. PLoS Pathog 5: e1000433. 19436712 10.1371/journal.ppat.1000433 PMC2674935

[48] Hessell AJ, Poignard P, Hunter M, Hangartner L, Tehrani DM, et al (2009) Effective, low-titer antibody protection against low-dose repeated mucosal SHIV challenge in macaques. Nat Med 15: 951–954. 19525965 10.1038/nm.1974 PMC4334439

[49] Gomez-Roman VR, Patterson LJ, Venzon D, Liewehr D, Aldrich K, et al (2005) Vaccine-elicited antibodies mediate antibody-dependent cellular cytotoxicity correlated with significantly reduced acute viremia in rhesus macaques challenged with SIVmac251. J Immunol 174: 2185–2189. 15699150 10.4049/jimmunol.174.4.2185

[50] Mabuka J, Nduati R, Odem-Davis K, Peterson D, Overbaugh J (2012) HIV-Specific Antibodies Capable of ADCC Are Common in Breastmilk and Are Associated with Reduced Risk of Transmission in Women with High Viral Loads. PLoS Pathog 8: e1002739. 22719248 10.1371/journal.ppat.1002739 PMC3375288

[51] Rerks-Ngarm S, Pitisuttithum P, Nitayaphan S, Kaewkungwal J, Chiu J, et al (2009) Vaccination with ALVAC and AIDSVAX to prevent HIV-1 infection in Thailand. N Engl J Med 361: 2209–2220. 19843557 10.1056/NEJMoa0908492

[52] Alter G, Moody MA (2010) The humoral response to HIV-1: new insights, renewed focus. J Infect Dis 202 Suppl 2: S315–322. 20846039 10.1086/655654 PMC2945610

[53] Haynes BF, Gilbert PB, McElrath MJ, Zolla-Pazner S, Tomaras GD, et al (2012) Immune-correlates analysis of an HIV-1 vaccine efficacy trial. N Engl J Med 366: 1275–1286. 22475592 10.1056/NEJMoa1113425 PMC3371689

[54] Gaschen B, Taylor J, Yusim K, Foley B, Gao F, et al (2002) Diversity considerations in HIV-1 vaccine selection. Science 296: 2354–2360. 12089434 10.1126/science.1070441

[55] Korber B, Gaschen B, Yusim K, Thakallapally R, Kesmir C, et al (2001) Evolutionary and immunological implications of contemporary HIV-1 variation. Br Med Bull 58: 19–42. 11714622 10.1093/bmb/58.1.19

[56] Trkola A, Matthews J, Gordon C, Ketas T, Moore JP (1999) A cell line-based neutralization assay for primary human immunodeficiency virus type 1 isolates that use either the CCR5 or the CXCR4 coreceptor. J Virol 73: 8966–8974. 10516002 10.1128/jvi.73.11.8966-8974.1999 PMC112928

[57] Howell DN, Andreotti PE, Dawson JR, Cresswell P (1985) Natural killing target antigens as inducers of interferon: studies with an immunoselected, natural killing-resistant human T lymphoblastoid cell line. J Immunol 134: 971–976. 3871222

[58] Yagita M, Huang CL, Umehara H, Matsuo Y, Tabata R, et al (2000) A novel natural killer cell line (KHYG-1) from a patient with aggressive natural killer cell leukemia carrying a p53 point mutation. Leukemia 14: 922–930. 10803526 10.1038/sj.leu.2401769

[59] Miller CJ, Genesca M, Abel K, Montefiori D, Forthal D, et al (2007) Antiviral antibodies are necessary for control of simian immunodeficiency virus replication. J Virol 81: 5024–5035. 17329327 10.1128/JVI.02444-06 PMC1900210

[60] O'Doherty U, Swiggard WJ, Malim MH (2000) Human immunodeficiency virus type 1 spinoculation enhances infection through virus binding. J Virol 74: 10074–10080. 11024136 10.1128/jvi.74.21.10074-10080.2000 PMC102046

[61] Alpert MD, Rahmberg AR, Neidermyer W, Ng SK, Carville A, et al (2010) Envelope-modified single-cycle simian immunodeficiency virus selectively enhances antibody responses and partially protects against repeated, low-dose vaginal challenge. J Virol 84: 10748–10764. 20702641 10.1128/JVI.00945-10 PMC2950576

[62] Frey A, Di Canzio J, Zurakowski D (1998) A statistically defined endpoint titer determination method for immunoassays. J Immunol Methods 221: 35–41. 9894896 10.1016/s0022-1759(98)00170-7

[63] National Research Council (1996) Guide for the Care and Use of Laboratory Animals. Washington DC: National Academy Press. pp 86–123.

[64] Marthas ML, Lu D, Penedo MC, Hendrickx AG, Miller CJ (2001) Titration of an SIVmac251 stock by vaginal inoculation of Indian and Chinese origin rhesus macaques: transmission efficiency, viral loads, and antibody responses. AIDS Res Hum Retroviruses 17: 1455–1466. 11679158 10.1089/088922201753197123 PMC3401017

[65] Salisch NC, Kaufmann DE, Awad AS, Reeves RK, Tighe DP, et al Inhibitory TCR coreceptor PD-1 is a sensitive indicator of low-level replication of SIV and HIV-1. J Immunol 184: 476–487. 19949078 10.4049/jimmunol.0902781 PMC2810496

[66] Cline AN, Bess JW, Piatak M Jr, Lifson JD (2005) Highly sensitive SIV plasma viral load assay: practical considerations, realistic performance expectations, and application to reverse engineering of vaccines for AIDS. J Med Primatol 34: 303–312. 16128925 10.1111/j.1600-0684.2005.00128.x

[67] Pal R, Venzon D, Letvin NL, Santra S, Montefiori DC, et al (2002) ALVAC-SIV-gag-pol-env-based vaccination and macaque major histocompatibility complex class I (A*01) delay simian immunodeficiency virus SIVmac-induced immunodeficiency. J Virol 76: 292–302. 11739694 10.1128/JVI.76.1.292-302.2002 PMC135699

[68] Mothe BR, Weinfurter J, Wang C, Rehrauer W, Wilson N, et al (2003) Expression of the major histocompatibility complex class I molecule Mamu-A*01 is associated with control of simian immunodeficiency virus SIVmac239 replication. J Virol 77: 2736–2740. 12552014 10.1128/JVI.77.4.2736-2740.2003 PMC141082

[69] Yant LJ, Friedrich TC, Johnson RC, May GE, Maness NJ, et al (2006) The high-frequency major histocompatibility complex class I allele Mamu-B*17 is associated with control of simian immunodeficiency virus SIVmac239 replication. J Virol 80: 5074–5077. 16641299 10.1128/JVI.80.10.5074-5077.2006 PMC1472056

[70] O'Connor DH, Mothe BR, Weinfurter JT, Fuenger S, Rehrauer WM, et al (2003) Major histocompatibility complex class I alleles associated with slow simian immunodeficiency virus disease progression bind epitopes recognized by dominant acute-phase cytotoxic-T-lymphocyte responses. J Virol 77: 9029–9040. 12885919 10.1128/JVI.77.16.9029-9040.2003 PMC167227

