# Supplementary figures and images for: ADCC Develops Over Time during Persistent Infection with Live-Attenuated SIV and Is Associated with Complete Protection against SIVmac251 Challenge
Source: PLoS Pathog. 2012 Aug 23;8(8):e1002890. doi: 10.1371/journal.ppat.1002890 (PMC3426556; doi:10.1371/journal.ppat.1002890)

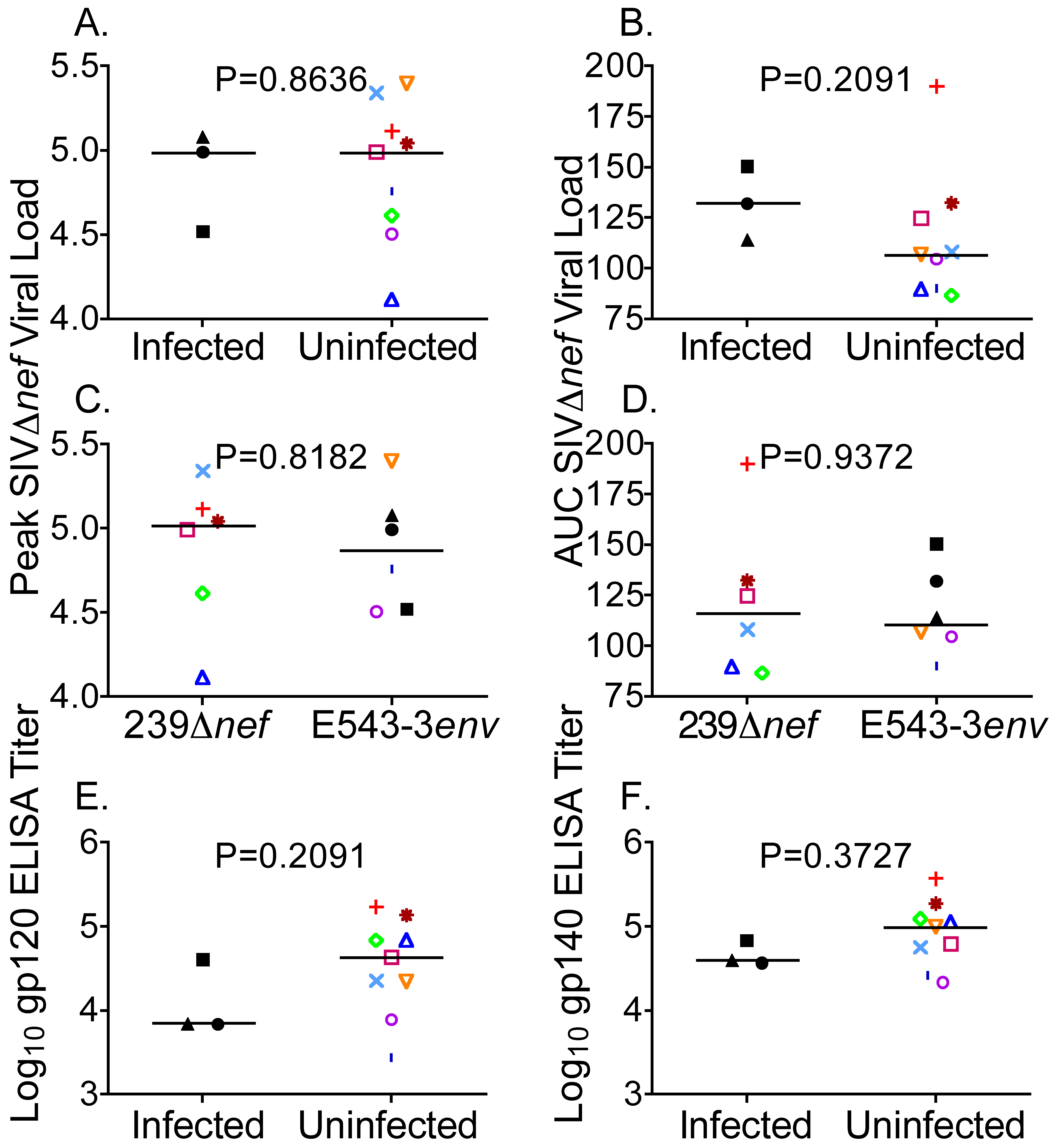

Supplement: Figure S1 — SIVΔ nef viral loads and ELISA titers among animals challenged with SIVmac251NE. There were no significant differences in vaccine strain viral loads among the animals that became infected versus those that remained uninfected after intravenous challenge with SIVmac251NE in terms of peak log10 RNA copies per ml (2-tailed Mann-Whitney U test, P = 0.8636) (A), or AUC log10 RNA copies per ml×weeks for the period of weeks 0–46 after inoculation (2-tailed Mann-Whitney U test, P = 0.2091) (B). SIVmac239Δnef and SIVmac239Δnef/E543-3env did not differ significantly in peak vaccine strain viral loads (2-tailed Mann-Whitney U test, P = 0.8182) (C), or in AUC values for vaccine strain viral loads over weeks 0–46 (P = 0.9372) (D). The outcome of challenge was not significantly related to binding antibodies measured by ELISA against recombinant SIVmac239 gp120 (2-tailed Mann-Whitney U test, P = 0.2091) (E), or gp140 (2-tailed Mann-Whitney U test, P = 0.3727) (F). (TIFF) [file ppat.1002890.s001.tiff]

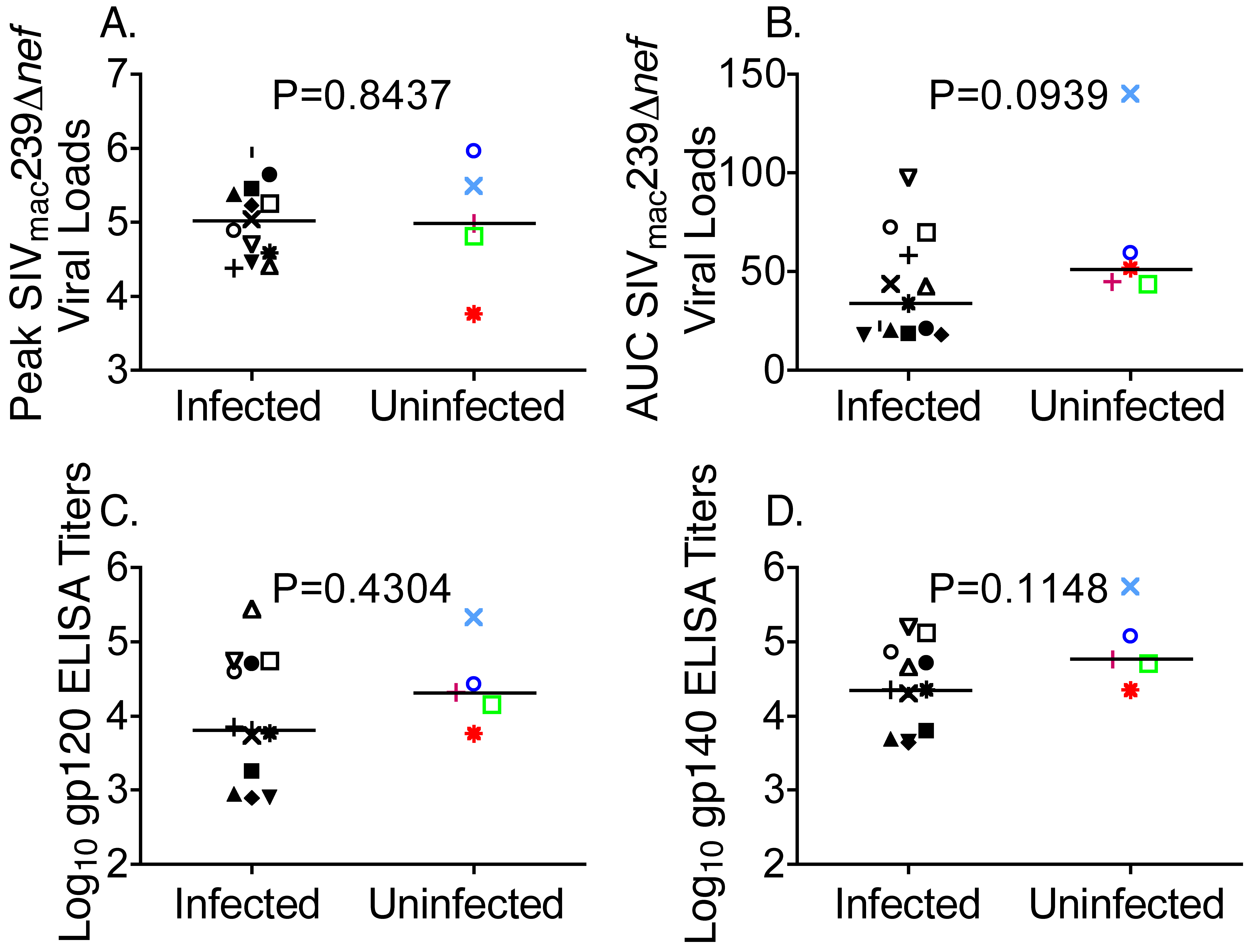

Supplement: Figure S2 — SIVmac239Δ nef viral loads and ELISA titers among animals challenged with SIVmac251UCD. Peak log10 SIVmac239Δnef viral loads appeared unrelated to protection against infection by SIVmac251UCD (2-tailed Mann-Whitney U test, P = 0.8437) (A). SIVmac239Δnef AUC log10 RNA copies per ml×weeks for the period through the day of challenge with SIVmac251UCD at 5, 20, or 40 weeks after inoculation appeared higher among the animals that remained uninfected, but this difference was not significant (2-tailed Mann-Whitney U test, P = 0.0939) (B). The SIVmac239Δnef-immunized animals that remained uninfected did not have significantly higher ELISA titers in sera collected on the day of challenge than those that became infected against SIVmac239 gp120 (2-tailed Mann-Whitney U test, P = 0.4304) (C), or gp140 (2-tailed Mann-Whitney U test, P = 0.1148) (D). (TIFF) [file ppat.1002890.s002.tiff]

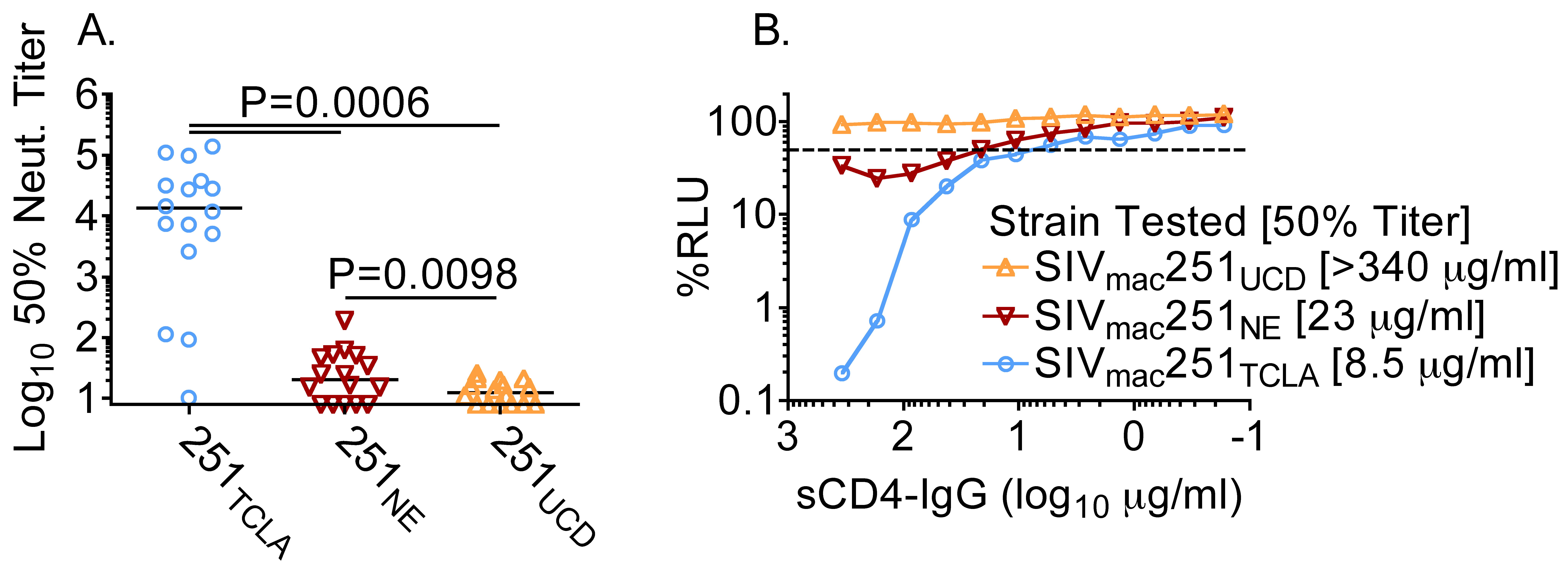

Supplement: Figure S3 — Neutralization resistance of SIVmac251 strains. SIVmac251NE, SIVmac251UCD, and T cell line-adapted SIVmac251TCLA were compared for their relative resistance to neutralization. Plasma samples from 16 macaques chronically infected with SIVmac239 were titered for neutralization of each strain (A). SIVmac251NE and SIVmac251UCD are both more resistant to neutralization by plasma than SIVmac251TCLA (2-tailed Wilcoxon matched pairs tests, P = 0.0006). SIVmac251UCD is more resistant to neutralization by plasma than SIVmac251NE (2-tailed Wilcoxon matched pairs test, P = 0.0098). SIVmac251NE, SIVmac251UCD, SIVmac251TCLA were also compared for their sensitivity to neutralization by a soluble CD4 protein, sCD4-IgG, which consists of human CD4 domains 1 and 2 fused to the IgG1 heavy chain (B). The dashed line indicates 50% of maximal infectivity. (TIFF) [file ppat.1002890.s003.tiff]
