# Supplementary material for: ADCC Develops Over Time during Persistent Infection with Live-Attenuated SIV and Is Associated with Complete Protection against SIVmac251 Challenge
Source: PLoS Pathog. 2012 Aug 23;8(8):e1002890. doi: 10.1371/journal.ppat.1002890 (PMC3426556; doi:10.1371/journal.ppat.1002890)
Supplement: Table S1 — Significance of differences among animals infected versus uninfected after SIVmac251UCD challenge. Differences among infected versus uninfected animals in peak SIVmac239Δnef viral loads, total SIVmac239Δnef replication (estimated from AUC values for log10 RNA copies per ml×weeks), gp120 ELISA titers, gp140 ELISA titers, 50% ADCC titers, and AUC values for ADCC were evaluated for significance by 2-tailed Mann-Whitney U tests. Significance was not determined (ND) for comparisons that included less than 3 animals in one group. (DOCX) [file ppat.1002890.s004.docx]

| **Table S1. Significance of differences among animals infected versus uninfected after SIV_mac_251_UCD_ challenge.** | | | | | |
| --- | --- | --- | --- | --- | --- |
|  |  |  |  |  |  |
|  | Time Points Included (Weeks) | | |  |  |
|  | 5 | 20 | 40 | 20, 40 | 5, 20, 40 |
| Peak SIVΔ*nef* Viral Load | ND | P=0.4 | ND | P=0.4318 | P=0.8437 |
| AUC SIVΔ*nef* Viral Load | ND | P=0.1 | ND | P=0.7551 | P=0.0939 |
| gp120 ELISA Titer | ND | P=0.7 | ND | P=0.8763 | P=0.4304 |
| gp140 ELISA Titer | ND | P=0.1 | ND | P=0.6389 | P=0.1148 |
| 50% ADCC Titer | ND | P=0.1 | ND | P=0.6389 | P=0.0487 |
| AUC Values for ADCC | ND | P=0.1 | ND | P=0.4318 | P=0.0761 |
|  |  |  |  |  |  |
